# Comparison of healing time of the 2^nd^ degree burn wounds with two dressing methods of fundermol herbal ointment and 1% silver sulfadiazine cream

**Published:** 2010

**Authors:** Reza Daryabeigi, Mohammad Heidari, Sayed Abbas Hosseini, Mahmoud Omranifar

**Affiliations:** * Department of Medical Surgical Nursing, School of Nursing and Midwifery, Isfahan University of Medical Sciences, Isfahan, Iran; ** School of Nursing and Midwifery, Isfahan University of Medical Sciences, Isfahan, Iran; *** Department of Fundamental Nursing, School of Nursing and Midwifery, Isfahan University of Medical Sciences, Isfahan, Iran; **** Associate Professor, Department of Surgery, School of Medicine, Isfahan University of Medical Sciences, Isfahan, Iran

**Keywords:** Burn wound, wound dressing, herbal ointment, sulfadiazine, wound healing

## Abstract

**BACKGROUND:**

Burn wounds are one of the health problems in modern societies that are associated with irreparable harms and many side problems for patients and their families. Infection due to burn wounds is the main cause of death in such patients. One of the methods to prevent infection of burn wounds is topical antibiotic ointments. This study aimed to investigate and identify effective ointments to treat burn wounds. For this purpose, the effects of two types of ointment, fundermol and 1% silver sulfadiazine cream on second degree burn wounds were compared.

**METHODS:**

This was a clinical trial study conducted in 2008. Using convenient and continuous sampling method, 50 patients referred to Imam Mousa Kazem Burn Injury Clinic in Isfahan, Iran with 2^nd^ degree burn wounds in 1% to 10% surface area were enrolled. The patients were randomly divided into two groups of treatment with fundermol and sulfadiazine and the dressing was changed once a day. The healing time for burn wounds in each patient was recorded in a checklist and data were analyzed by independent t-test via SPSS software.

**RESULTS:**

The healing time of burn wounds in the group treated with fundermol was shorter than that in the group treated with sulfadiazine (p < 0.001).

**CONCLUSIONS:**

The present study showed that fundermol ointment accelerates burn wound healing. Therefore, fundermol can be introduced as a good replacement for current treatments of burn wounds.

Burn wounds are one of the health problems in modern societies that are associated with irreparable harms and many side problems for patients and their families. Burning is a disaster in human society, a painful death and an incident full of wounds and injury that can darken the beautiful image of life at once and emerge ugly shadows of pain and sorrow.^1^ According to statistics, about 2.5 millions of Americans suffer from burn injuries every year, of which about 100,000 are admitted to hospitals. More than 10,000 of these patients lose their lives due to complications of burn injuries and it has the highest mortality rate due to driving accidents.^2^ On the other hand, developments of recent decades in health care and treatment of patients with burns have increased life expectancy and reduced mortality rate in these patients.^3^ Studies showed that burn infection is the main cause of mortality in patients with extensive burns.^4^ Therefore, many researchers tried to achieve appropriate treatment methods to reduce the risk of wound infections and to shorten the period of treatment of patients with burn wounds. Some of these treatments involve using topical antimicrobial agents which effectively reduce mortality rate of burns.^5^ One of these antimicrobial topical ointment is 1% silver sulfadiazine, with advantages such as easy and convenient use, not to create pain when consumed, yielding low toxicity and sensitivity and having anti-bacterial effect, which made it known as the gold standard of anti-microbial topical drugs for patients with burns^6^ and turned it to the main consumed medicine in treatment of burn wounds around the world.^7^ However, studies have shown that this drug also causes side effects such as reduction of white blood cells, toxic epidermal necrolysis, increased skin pigmentation, neutropenia, increased bacterial resistance and the skin appearance would not return to normal.^8^ Another problem of this medicine in Iran is related to unavailability of its raw material which should be imported and consequently makes its price very high for patients.^9^ Considering the physical, financial, emotional and psychological complications imposed on burn injured patients and their families as well as the health system, and also the low level of facilities in Iranian health centers, it is necessary to pay special attention to outpatient treatment not only to reduce medical expenses resulting from hospitalization, but to provide health services to more patients with burn wounds.^10^ Therefore, making new drugs that can control burn wound infection, its related shock and other aspects of burn wound with less complications and can accelerate the wound healing and reduce the mortality of patients is one of the research priorities in Iran.^11^ For this purpose, a new ointment called fundermol that is completely herbal and its raw materials are supplied within the country was made in Iran. In recent years, this ointment has been used for the treatment of relatively thick burns. Its effective substance is lawsone (2 hydroxy 1,4-naphthoquinone) derived from Henna (Hina) or *Lawsonia inermis*. Lawson is auinone derivative and increases tendency of oxygen combination with red blood cells and strengthens red blood cell membrane. In addition, it has anti-bacterial and anti-fungal effects. Basis of this drug is bee wax.^12^ Given the fact that dressing is duty of nurses, the role of nurses in dressing and caring of burn wounds is a key effective role. Nurses can significantly reduce the amount of physical, financial, emotional and psychological harms imposed on burn injured patients by following health prin-ciples and educating patients for as best possible as care of the burned area. Considering the point that observation is the basis of care and also given the limitations of using 1% silver sulfadiazine cream and the bacterial resistance created to this medicine and considering that all studies conducted so far on the effect of fundermol have been in laboratory conditions and the obtained results of these studies have shown the effectiveness of this medicine on healing burn wounds, the present study was conducted to investigate and compare the effect of fundermol and 1% silver sulfadiazine cream on healing 2^nd^ degree burn wounds of patients referred to Imam Mousa Kazem Burn Injury Clinic in Isfahan, Iran.

## Methods

This was a clinical trial study conducted in 2008. Sampling was simple and continuous and 50 patients who referred to Imam Mousa Kazem Burn Injury Clinic with 2^nd^ degree burn wounds in 1 to 10 percents surface area were enrolled. Entry criteria included age of 2-60 year-old, burn wounds following contact with heaters or hot liquids, referred to the clinic in less than 6 hours after burn incidence, normal amount of hemoglobin and total protein in patients, and no background disease such as anemia, diabetes, cardiovascular diseases, cancer, and immune system defects. After the type and the percentage of burn were determined and no requirement to hospitalization was approved by the physician of the health clinic, the patients were randomly divided into two groups of treatment with fundermol and 1% silver sulfadiazine. Patients with odd numbers were put in fundermol group and those with even numbers were put in sulfadiazine group. Patients in fundermol group and sulfadiazine group received dressing with fundermol herbal ointment and 1% silver sulfadiazine cream, respectively. The medicine was prescribed by physicians and patients of both groups received oral antibiotics for preventive reasons. Dressing method for fundermol group included washing with sterile saline 0.9 percent, leaving it for 5 minutes in fresh air so that the wound surface was completely dried. Then, using sterilized absolang size 1 mm, fundermol ointment was applied on the wound and the wound was covered by Nailex. The dressing in group sulfadiazine included washing the burn wounds surface with sterile saline 0.9 percent and leaving it for 5 minutes in fresh air; so that the wound surface was completely dried. Then, using sterilized absorbant, 1% silver sulfadiazine cream was applied on the wound with 3 mm thickness and a light dressing of sterile gauze was placed on it. Wound dressings were changed daily until complete recovery. The wounds considered healed when it turned into bright light pink color without any secretion and with a coating tissue. It should be mentioned that all patients were completely aware of the type of medicine used on their wounds and they completed a written consent form. Also, the ethics committee of Isfahan University of Medical Sciences approved the research. Finally, the time of referral and the time of healing were recorded and the mean healing time of the two groups was determined and compared by independent sample t-test. The data were analyzed by SPSS software.

## Results

Data analysis revealed that about 74% of patients referred to the health center were men. Also, about half of the patients in both genders were in the age range of 21 to 42 year-old.

The data also showed that most organ injuries have been in upper limbs, lower limbs and torso, respectively; and hot liquid was also the most common cause of burns and after that, flame and hot objects were the common factors in causing burn wounds in patients.

The mean time of healing in fundermol group was 4.4 days with standard deviation of 1.87 days while the mean healing time for 1% silver sulfadiazine group was 5.9 days with standard deviation of 2.20 days. Independent t-test showed that their difference was statistically significant (p = 0.014).

## Discussion

According to our findings, the burning was more prevalent in men. The higher burn incidence among men can be due to their career and job, since they contact with burning substances and fire more than women.

Burn is a physical and chemical phenomenon and the cause of many morbidities and mortalities in the world. The final goal of all current treatments of burn was to accelerate skin healing and prevent wound infection.^13^

Previous studies have focused on the effects of fundermol ointment on process of healing burn wounds in laboratory conditions. Bagheri Yazdi et al studied the effects of fundermol ointment on healing 2^nd^ degree burn wounds in rats and found that fundermol ointment was effective in providing tensile strength in healing tissue and also, blood vessel generation in the area of burn wound; it accelerated the process of wound healing.^14^ Rastgar Lari et al compared the 1% silver sulfadiazine and fundermol ointment on rats and found that fundermol in healing burn wounds with relative thickness is more effective than 1% silver sulfadiazine.^12^ Recently, Hosseini et al examined the effects of fundermol and sulfadiazine on healing second degree burn wounds with infection by pseudomonas in experimental animals. The results showed that fundermol ointment in the treatment of burn wounds was more effective than 1 percent silver sulfadiazine. Also, fundermol ointment controlled the infection better, reduced the possibility of overlay burn scar in the burn wound area and the economic cost was also lower.^9^ The results of the present study showed that fundermol ointment was more effective than 1 percent silver sulfadiazine; given its merits such as shorter healing time and recovering the injured tissue, reducing movement limitations due to using nailex in dressing with this ointment, reducing treatment costs and its easy access, in addition to being produced locally (all the ingredients are local) and reducing import needs, fundermol ointment can be considered an appropriate replacement for current treatments of burn wounds.

Introducing this medicine to health care personnel, specially physicians and nurses, who have a key role in treatment and providing health care to patients suffer from burn wounds led this medicine to be more applicable in clinical centers. In addition to shortening the duration of hospitalization for such patients, the imposed costs of health care can be reduced. Considering the limitation of facilities and health clinics for burn wounds in Iran, health services can be provided to more patients by this way.

It should be mentioned that clinical researches on this medicine are few. It is recommended that further studies examine its effects on healing burn wounds of more extensive areas.

The authors declare no conflict of interest in this study.

**Table 1:** Frequency distribution of genders and its comparison between the two groups of silver sulfadiazine and fundermol

Gender	Fundermol		1% Silver Sulfadiazine		Total		χ^2^	p
			
	Number	Percent	Number	Percent	Number	Percent		
Male	18	72	19	76	37	74		
Female	7	28	6	24	13	26	0.104	0.747
Total	25	100	25	100	50	100		

**Table 2:** Frequency distribution of burn areas and its comparison in the two groups of fundermol and sulfadiazine

Burn area	Fundermol		1% Silver Sulfadiazine		Total		χ^2^	p
			
	Number	Percent	Number	Percent	Number	Percent		
Upper limbs	19	76	14	56	33	66		
Lower limbs	4	16	8	32	12	24		
							2.291	0.318
Torso	2	8	3	12	5	10
Total	25	100	25	100	50	100		
